# Humanized Rag1^−/−^γc^−/−^ Mice Support Multilineage Hematopoiesis and Are Susceptible to HIV-1 Infection via Systemic and Vaginal Routes

**DOI:** 10.1371/journal.pone.0020169

**Published:** 2011-06-14

**Authors:** Ramesh Akkina, Bradford K. Berges, Brent E. Palmer, Leila Remling, C. Preston Neff, Jes Kuruvilla, Elizabeth Connick, Joy Folkvord, Kathy Gagliardi, Afework Kassu, Sarah R. Akkina

**Affiliations:** 1 Department of Microbiology, Immunology, and Pathology, Colorado State University, Fort Collins, Colorado, United States of America; 2 Department of Medicine, University of Colorado Denver, Aurora, Colorado, United States of America; George Mason University, United States of America

## Abstract

Several new immunodeficient mouse models for human cell engraftment have recently been introduced that include the Rag2^−/−^γc^−/−^, NOD/SCID, NOD/SCIDγc^−/−^ and NOD/SCIDβ2m^−/−^ strains. Transplantation of these mice with CD34^+^ human hematopoietic stem cells leads to prolonged engraftment, multilineage hematopoiesis and the capacity to generate human immune responses against a variety of antigens. However, the various mouse strains used and different methods of engrafting human cells are beginning to illustrate strain specific variations in engraftment levels, duration and longevity of mouse life span. In these proof-of-concept studies we evaluated the Balb/c-Rag1^−/−^γ^−/−^ strain for engraftment by human fetal liver derived CD34^+^ hematopoietic cells using the same protocol found to be effective for Balb/c-Rag2^−/−^γc^−/−^ mice. We demonstrate that these mice can be efficiently engrafted and show multilineage human hematopoiesis with human cells populating different lymphoid organs. Generation of human cells continues beyond a year and production of human immunoglobulins is noted. Infection with HIV-1 leads to chronic viremia with a resultant CD4 T cell loss. To mimic the predominant sexual viral transmission, we challenged humanized Rag1^−/−^γc^−/−^ mice with HIV-1 via vaginal route which also resulted in chronic viremia and helper T cell loss. Thus these mice can be further exploited for studying human pathogens that infect the human hematopoietic system in an in vivo setting.

## Introduction

Humanized mice constructed by engrafting human tissues/cells into immunodeficient mice have greatly advanced research with viruses such as HIV since human target cells are provided in a physiological setting thus permitting the study of human disease pathogenesis, immunity and testing of antivirals in vivo [1], [2], [3]. While the original SCID-hu thy/liv mouse and SCID-Hu-PBL models have been very useful to a certain extent in this context there is no de novo multilineage hematopoiesis with full complement of all the immune system cells [4], [5], [6]. The CB17 SCID mice (Prkdc mutation) can spontaneously generate murine T and B cells as they age (referred to as “leakiness”) and have high levels of NK cell activity, both of which prevent efficient and prolonged xenoengraftment [7]. Further, the Prkdc mutation contributes to increased radiosensitivity due to a defect in DNA repair. Thus, the irradiation step that is sometimes used to condition the mice for exogenous cell engraftment leads to stunted growth and decreased life span.

Recent advances in the derivation of newer immunodeficient mouse strains have permitted improved human cell engraftment with human cells such as CD34^+^ hematopoietic progenitor cells [1], [8]. A variety of mutations are responsible for the SCID (severe combined immunodeficiency syndrome) phenotype with a deficiency in different lymphoid cell populations [1]. The mutations fall into two basic categories: in genes required for the production of T and/or B cell receptors and genes required for the response to cytokines involved in the lymphoid cell maturation/proliferation and interactive communication. The first category includes Prkdc (protein kinase DNA-activated catalytic polypeptide), adenosine deaminase (ADA), Janus kinase-3 (JAK3), Artemis, and the two Rag (recombination-activating gene) proteins, Rag1 and Rag2. Artemis is an endonuclease involved in the DNA recombination event required to generate T and B cell receptors. Rag1 and Rag2 proteins form a complex with DNA to configure a hairpin structure necessary for the endonuclease activity of Artemis. Rag1^−/−^ and Rag2^−/−^ mice phenotypes are similar [9], [10]. No leakiness or radiosensitivity is associated with either Rag1 or Rag2 mutations as is commonly seen in *Prkdc^−/−^* mice. The second common category of mutations leading to immunodeficiency is the lack of the common gamma chain (γc) which is an integral part of receptors required for the response to the cytokines IL-2, IL-4, IL-7, IL-9, IL-15, and IL-21. This leads to a failure in various cell types to mature and/or expand, including T cells, B cells, and natural killer (NK) cells [11], [12], [13]. Other mutations resulting in decreased NK cell activity (e.g., non-obese diabetic or NOD) have also been shown to support improved xenoengraftment [14]. However, a disadvantage with the NOD strain is high incidence of lymphomas leading to a shortened lifespan.

Exploitation of these above new generation immunodeficient mice leads to improved humanized mice with higher and more sustained human cell engraftment. These include mouse strains such as Rag2^−/−^γc^−/−^, NOD/SCID, NOD/SCIDγc^−/−^, and NOD/SCIDγ2m^−/−^ mice [1], [8], [15], [16], [17]. Transplantation with human CD34 hematopoietic stem cells resulted in de novo multilineage human hematopoiesis with the generation of T cells, B cells, macrophages and dendritic cells which constitute the main players in an adaptive immune response. Human cells were shown to populate the primary and secondary lymphoid system organs.

The new and improved humanized mice provided new opportunities in the study of human pathogens that infect the hematopoietic system. To date, the newer humanized mouse strains have been evaluated for susceptibility to infection with a variety of human viruses including EBV, HIV-1, HTLV-1, and dengue virus to name a few [8], [18], [19], [20], [21]. Human T cell responses have been demonstrated against HIV-1, EBV, toxic shock syndrome toxin 1, dengue virus and a recombinant adenoviral vector expressing HCV proteins [8], [22], [23], [24], while human antibody responses have been shown against HIV-1, dengue virus, tetanus toxoid, and the haemophilus influenzae B conjugate vaccine [8], [18], [20], [24], [25], [26]. Evidence for antigen-specific antibody class-switching and the detection of neutralizing antibody following dengue infection attests to the potency of the adaptive immune response generated in these new and improved models [18]. Human T and B cell receptors in humanized mice were found to be highly diverse [23]. Furthermore, mucosal engraftment is also seen with human cells detected in gut, rectal and vaginal mucosa [27], [28], [29], [30], [31], [32], [33]. Indeed, efficient HIV-1 transmission via both vaginal and rectal routes was demonstrated in RAG-hu and BLT mice which lead to successful evaluation of new pre-exposure chemo-prophylactics to prevent HIV-1 sexual transmission [27], [29], [34].

Despite the above advances, some shortcomings still exist and newer strains of mice for better hematopoietic cell engraftments are continually sought. Other important criteria for efficient production of humanized mice are litter size, robustness after irradiation and general health issues that may negatively impact the overall experimentation with this system. With these as a background, here we characterized a new mouse strain for engraftment of human CD34^+^ hematopoietic stem cells, namely the Balb/c-Rag1^−/−^γc^−/−^ mouse. We demonstrate efficient human cell engraftment and multilineage hematopoiesis using a similar protocol we previously employed for engraftment of Balb/c-Rag2^−/−^γc^−/−^ mice (neonatal gamma irradiation followed by intrahepatic injection of fetal liver-derived human CD34^+^ cells) [19]. Human hematopoietic cells were found to populate different lymphoid organs and there was production of human immunoglobulins IgM, IgG, and IgA. We also show that humanized Rag1^−/−^γc^−/−^ mice are susceptible to HIV-1 infection, thus demonstrating that these mice are useful for the study of human viral pathogenesis. In addition, we also compared the general aspects of overall breeding characteristics and survival rates between Rag1^−/−^γc^−/−^ and Rag2^−/−^γc^−/−^ mice.

## Materials and Methods

### Generation of humanized Rag1^−/−^γc^−/−^ mice (RAG1-hu mice)

Balb/c-Rag1^−/−^γc^−/−^ mice (hitherto referred to as BALB/c-Rag1^−/−^γc^−/−^ mice or simply RAG1 mice) were derived and custom bred (C.129S4IL2RG×C.129S7 Rag1) at the Jackson Laboratories (Bar Harbor, ME) through a paid contract. Humanized mice were prepared by engraftment with human fetal liver-derived CD34^+^ hematopoietic progenitor cells as we previously described for the generation of humanized BALB/c- Rag2^−/−^γc^−/−^ mice [19]. Mice were maintained at the Colorado State University Painter Animal Center. These studies have been reviewed and specifically approved by the CSU Institutional Animal Care and Use Committee (Protocol 09-1460A). Briefly, newborn mice were conditioned by irradiating with 350 rads and then injected intrahepatically with 0.5–1×10^6^ human CD34^+^ cells. Mice were screened for human cell engraftment at 12 weeks post-reconstitution. Peripheral blood was collected by tail bleed and red blood cells were lysed by using the Whole Blood Erythrocyte Lysing Kit (R&D Systems, Minneapolis, MN). The white blood cell fraction was stained with antibodies against the human pan-leukocyte marker CD45 (Caltag) and FACS analyzed to determine the levels of human cell engraftment as we previously described [19]. For vaginal infections with HIV, female mice with over 40% engraftment were used.

### Flow cytometry

Whole blood was collected and red blood cells lysed as reported previously [19], [27]. White blood cells were stained with CD45-PE, CD3-PE, CD4-PECy5, CD8-PE, CD14-PE, (Caltag), CD19-PE, CD123-PE (eBioscience) and CD20-FITC, CD11c-PE-Cy5 (BD Pharmingen). To evaluate the presence of human hematopoietic cells in different mouse organs, single cell suspensions were made from spleen, thymus, lymph nodes, liver and bone marrow. Cells were stained with different labeled antibodies and subjected to multiparametric FACS analysis. Stained cells were analyzed using a Coulter EPICS XL-MCL FACS analyzer (Beckman Coulter, Fullerton, CA) or BD LSRII. To determine CD4 T cell loss in HIV-1 infected mice, they were bled weekly and CD4^+^ T cell levels were calculated as a ratio of the entire CD3 population (CD4^+^CD3^+^∶CD4^−^CD3^+^) as described previously [19]. To establish baseline CD4^+^ T cell ratios, all mice were analyzed prior to infection.

### Immunohistochemistry

Immunohistochemistry for human lymphocytes and macrophages was performed on 4 mm frozen OCT tissue sections. Primary antibodies used were anti-human CD45 LCA (clone 2B11+PD7/26, DakoCytomation, Denmark), anti-human CD3 (clone UCHT1, BD Pharmingen), anti-human CD4 (clone RPA-T4, BD Pharmingen), anti-human CD8 (clone HIT8A, BD Pharmingen), and anti-human CD68 (clone EBM11, DakoCytomation). Staining was performed as reported previously using the Vector Laboratories Mouse on Mouse staining kit [19].

### Measurement of total human antibody production

Total human antibody production was measured by ELISA using the Total Human IgG/IgM/IgA Assays (ALerCHEK, Portland, ME). Serum samples were analyzed following manufacturer's protocol. Secretory IgA was measured as follows: 50 ul sterile 1× PBS was introduced into vaginal vault with a P200 pipet tip smoothed by briefly passing over a flame. PBS was pipetted back and forth to collect the vaginal washings. On some occasions, mucus plugs were visible. To solubilize mucus, samples were incubated at 37°C for 30 min prior to ELISA testing.

### HIV-1 infection and measurement of viral loads

Mice were infected with HIV-1 by intraperitoneal injection of 100 ul of strain NL4-3 (1.2×10∧5 iu) or 100 ul of strain BaL-1 (0.9×10∧5 iu). Vaginal infections were performed in a volume of 25 ul with BaL-1 virus (3000 TCID). Sterile P200 tips that had been previously heated over a flame to smooth any abrasive surfaces were used to deliver the virus [27]. Anesthetized mice were held in an inverted position for four minutes post-inoculation to allow virus to adsorb and to prevent immediate discharge of virus. Mice were observed daily and blood samples drawn weekly to assess plasma viremia. To detect HIV-1 in plasma of infected mice by RT-PCR, RNA was extracted from 25–50 ul of EDTA-treated plasma using the QIAamp Viral RNA kit (Qiagen, Valencia, CA). Q-PCR was performed using a primer set specific for the HIV-1 LTR sequence and a corresponding LTR specific probe as described previously [19].

## Results

### Rag1^−/−^γc^−/−^ mice permit efficient human CD34^+^ hematopoietic cell engraftment

**Figure 1:**
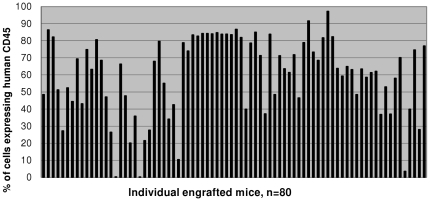
Human CD45 cell engraftment levels in humanized Rag1^−/−^γc^−/−^ mice. Human CD34 cell reconstituted mice were bled at 12 weeks post-engraftment. RBCs were lysed and the white blood cell fraction was stained for human panleukocyte CD45 marker and FACS analyzed. The level of human cell engraftment for each mouse is depicted.

Neonatal Rag1^−/−^γc^−/−^ mice were transplanted with human fetal liver derived CD34^+^ cells after conditioning by irradiation as we described previously to prepare humanized Rag2^−/−^γc^−/−^ mice [19]. At twelve weeks post-reconstitution, mice were screened for human cell engraftment in peripheral blood by staining for human CD45 marker followed by FACS analysis. Summary of CD45 cell engraftment levels for eighty humanized Rag1^−/−^γc^−/−^ mice are presented in Fig. 1. The mean peripheral blood human cell engraftment was 59.3+/−2.5% [SEM], with 41% of mice showing at least 70% engraftment and 5% of mice with less than 20% engraftment. In comparison, humanized Rag2^−/−^γc^−/−^ mice similarly reconstituted with CD34 cells had a mean peripheral blood engraftment of 48.3+/−3.0% (data not shown). The higher peripheral blood engraftment seen in humanized Rag1^−/−^γc^−/−^ mice over Rag2^−/−^γc^−/−^ mice is statistically significant (unpaired t test, P = 0.005, n = 100 each).

### Rag1^−/−^γc^−/−^ mice support multilineage human hematopoiesis and human cells populate different lymphoid organs

**Figure 2:**
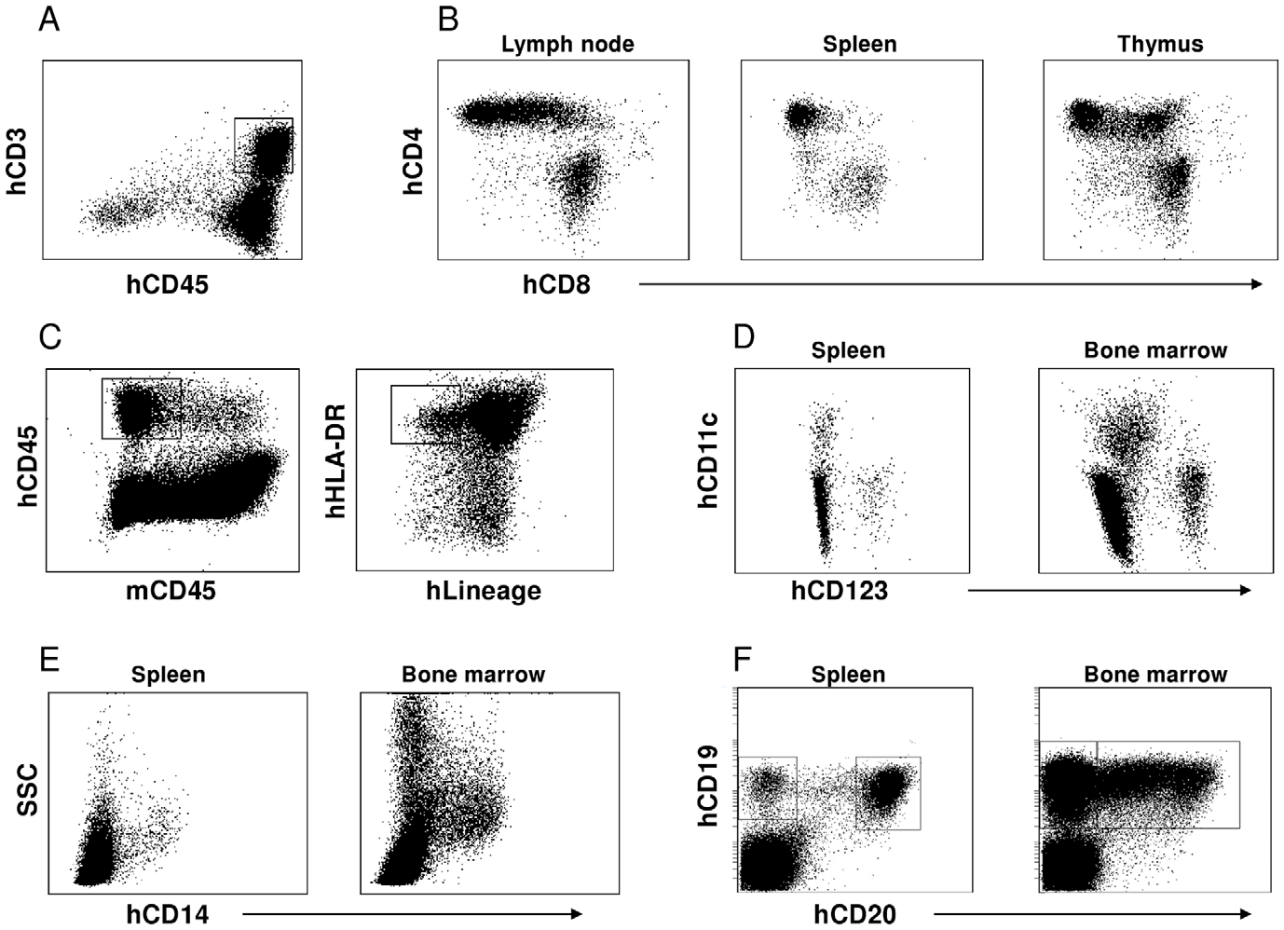
FACS analysis of multilineage human hematopoiesis in humanized Rag1^−/−^γc^−/−^ mice. Single cell suspensions were made from spleen, bone marrow, lymph node and thymus of humanized Rag1^−/−^γc^−/−^ mice and were stained with different antibodies to detect human hematopoietic cell sub-sets. Human anti-CD45 antibody was used to define human leukocytes. From this population human CD3+ T cells (A) as well as CD4+ and CD8+ T cell subsets were identified in the lymph node, spleen and thymus (B). Dendritic cells were identified by their lack of lineage staining (CD3, CD19, CD14, CD16, CD20 and CD56) and expression of HLA DR (C). Both myeloid (CD11c) and plasmacytoid (CD123) dendritic cells (D) were identified in lymphoid organs as were CD14 expressing monocytes (E) and two subsets of CD19 and CD20 expressing B cells (F).

**Figure 3:**
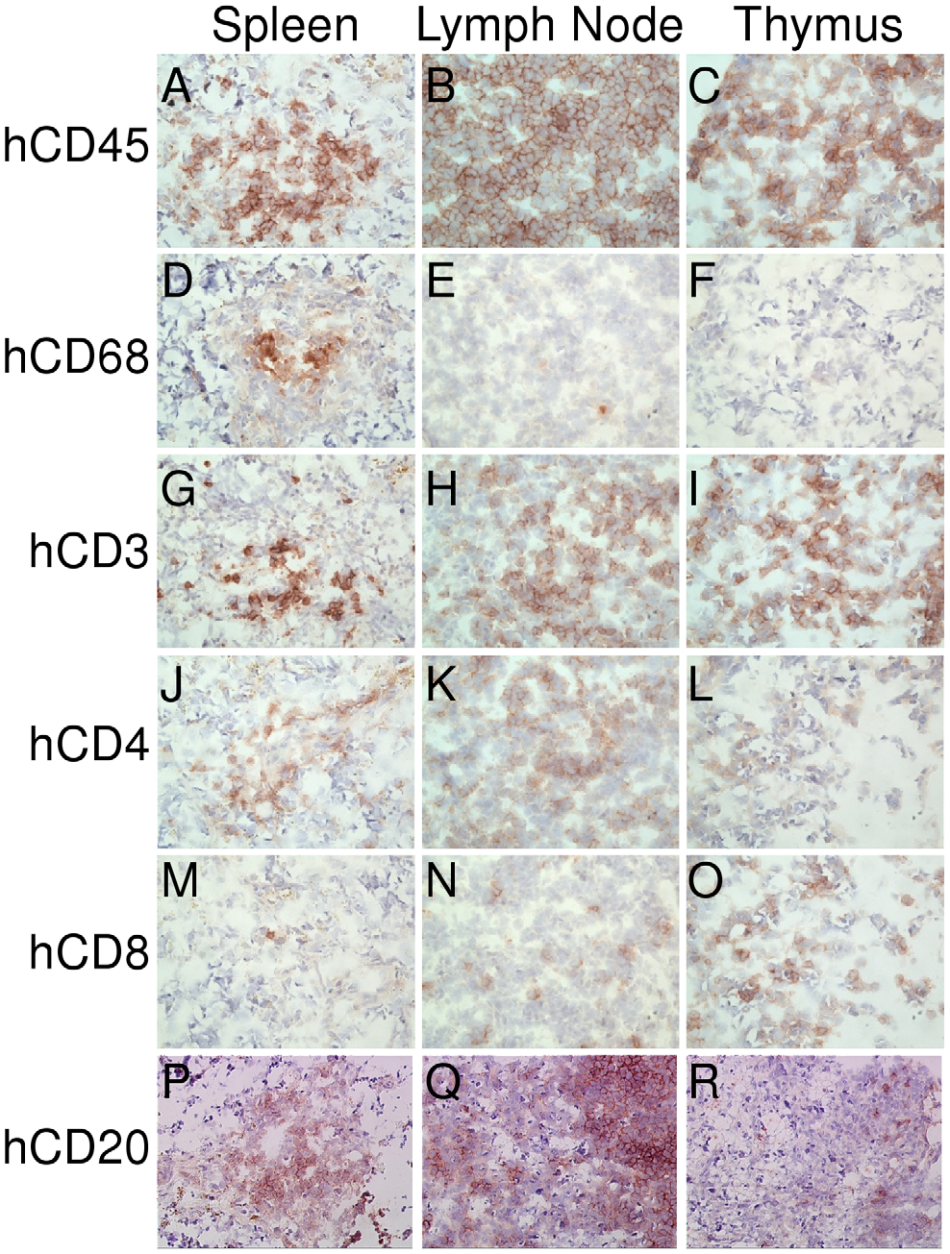
Immunohistochemical staining of human hematopoietic cells in lymphoid organs. Tissue sections of lymphoid organs (spleen, lymph node, and thymus) from humanized Rag1^−/−^γc^−/−^ mice were stained for the presence of human leukocytes. CD45^+^ leukocytes (A,B,C), CD68^+^ macrophages/dendritic cells (D,E,F), CD3^+^ T cells (G,H,I), CD4^+^ helper T cells (J,K,L), CD8^+^ cytotoxic T cells (M,N,O) and CD20^+^ B cells (P,Q,R) were detected in each of the organs assayed.

After determining the peripheral blood engraftment with human cells, we next proceeded to evaluate the presence of different lymphoid and myeloid cell sets in various organs by multiparametric flow cytometry and immuno-histological staining (Figs. 2 and 3). Human CD45+ hematopoietic cells were found in spleen, bone marrow lymph node and thymus of engrafted mice. Human CD3+CD4+ and CD3+CD8+ T cells were detected in the spleen, lymph node and thymus. As expected, there was about twice as many CD4+ T cells as CD8+ T cells in all organs. Consistent with ongoing de novo lymphopoiesis, CD4/CD8 double positive immature thymocyte population was seen in the thymus (Fig. 2B). Using a lineage cocktail antibody panel containing monoclonal antibodies directed against B cells, T cells, monocytes, NK cells and HLA-DR, dendritic cells were identified as human CD45 positive, lineage negative and HLA-DR positive (Fig. 2C). Furthermore, distinct populations of CD123+ cells, (plasmacytoid dendritic cells i.e., pDCs) and CD11c+ cells (myeloid dendritic cells i.e., mDCs) were detected in the spleen and bone marrow, demonstrating the presence of the two main dendritic cell subsets (Fig. 2D). Monocytes expressing CD14 (Fig. 2E) as well as CD19^+^CD20^+^ B cells (Fig. 2F) were also detected in both bone marrow and spleen.

Immunohistochemistry was performed to detect human cell types in situ in spleen, mesenteric lymph nodes, and thymus (Fig. 3). Human CD45^+^ cells were readily detectable in all the three organs (Fig. 3A–C), while only rare CD68^+^ macrophages stained positive (Fig. 3D–F). CD3^+^ T cells were also found in all three organs (Fig. 3G–I), as well as the CD4^+^ (Fig. 3J–L) and CD8^+^ T cell subsets (Fig. 3M–O). CD20^+^ B cells were also detected in all three organs (Fig. 3P–R).

### Humanized Rag1^−/−^γc^−/−^ mice generate human immunoglobulins

**Figure 4:**
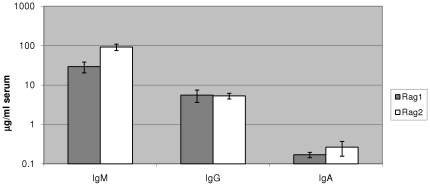
Human antibody production in humanized Rag1^−/−^γc^−/−^ mice. Blood was drawn at 16 weeks post human CD34 cell engraftment and sera were analyzed by ELISA to detect different human immunoglobulin classes.

Since all the necessary hematopoietic cell subsets involved in humoral immune response are present in engrafted Rag1^−/−^γc^−/−^ mice, they are expected to generate human antibodies. Accordingly, we looked for the presence of human immunoglobulins IgM, IgG, and IgA in these mouse sera 16 weeks post-CD34 cell engraftment. Immunoglobulin specific ELISA was used to determine any quantitative differences when compared with Rag2^−/−^γc^−/−^ mice (Fig. 4). The mean serum concentration of human IgM was 29.4+/−9.0 µg/ml and 92.3+/−17.0 µg/ml in humanized Rag1^−/−^γc^−/−^ and Rag2^−/−^γc^−/−^ mice, respectively. The mean serum concentration of human IgG was 5.6+/−1.9 and 3.3+/−0.9 µg/ml in Rag1^−/−^γc^−/−^ and Rag2^−/−^γc^−/−^ mice, respectively. The mean serum concentration of human IgA was 0.17+/−0.03 and 0.26+/−0.11 µg/ml in Rag1^−/−^γc^−/−^ and Rag2^−/−^γc^−/−^ mice, respectively. The difference in serum IgM level was significant (unpaired t test, P = 0.003; n = 18 Rag1^−/−^γc^−/−^ and n = 20 Rag2^−/−^γc^−/−^). We also determined if secretory antibodies exist in the mucosal sites of these mice. Vaginal washes were subjected to ELISA to detect the levels of secretory IgA antibody (see Methods). Of the 4 Rag1^−/−^γc^−/−^ mice assayed, two were positive for IgA in the vaginal mucus (0.19 and 0.15 µg/ml) whereas only one was positive among the 10 Rag2^−/−^γc^−/−^ mice tested (0.11 µg/ml). It should be noted that the lone Rag2^−/−^γc^−/−^ mouse that was positive for sIgA had a serum IgA concentration that was nearly five-fold higher than any other mice tested.

### Relative yields of humanized Rag1^−/−^γc^−/−^ mice versus Rag2^−/−^γc^−/−^ mice

To evaluate the practical aspects of generation of humanized mice, we compared the Rag1^−/−^γc^−/−^ mice to Rag2^−/−^γc^−/−^ mice. We found that two factors led to higher yields of humanized Rag1^−/−^γc^−/−^ mice (Table 1). First, Rag1^−/−^γc^−/−^ mouse litters had 20% more pups per litter than Rag2^−/−^γc^−/−^ mice (4.35 and 3.62 pups/litter, respectively) with a statistical significance (unpaired t test, P = 0.022; n = 80 Rag1^−/−^γc^−/−^ litters and n = 76 Rag2^−/−^γc^−/−^ litters). We also observed that Rag1^−/−^γc^−/−^ pups survived the engraftment protocol better than Rag2^−/−^γc^−/−^ pups (survival to screening at 12 weeks post-engraftment), with 48% higher survival of humanized Rag1^−/−^γc^−/−^ mice (79.6% Rag1^−/−^γc^−/−^ survival and 53.7% Rag2^−/−^γc^−/−^ survival, respectively). The difference was again significant (unpaired t test, P = 0.004; n = 54 litters each). The net effect of larger litters together with better survival after the engraftment protocol resulted in 78.4% higher yields of humanized Rag1^−/−^γc^−/−^ mice as compared to Rag2^−/−^γc^−/−^ mice.

**Table 1:** Comparison of Rag1^−/−^γc^−/−^ versus Rag2^−/−^γc^−/−^ mice for generation of humanized mice (Hu Mice).

	Rag2^−/−^γc^−/−^	Rag1^−/−^γc^−/−^	Comments:Rag1^−/−^γc^−/−^ vs Rag2^−/−^γc^−/−^
Litter size	3.62 pups	4.35 pups	20.2% more pups
Survival levels at 12 wk engraftment screening	53.7%	79.6%	48.2% higher survival
Average yieldHu Mice/litter	1.94	3.46	78.4% more Hu Mice

### Humanized Rag1^−/−^γc^−/−^ mice are permissive to HIV-1 infection via intra-peritonial and vaginal mucosal routes and show CD4 T cell loss

**Figure 5:**
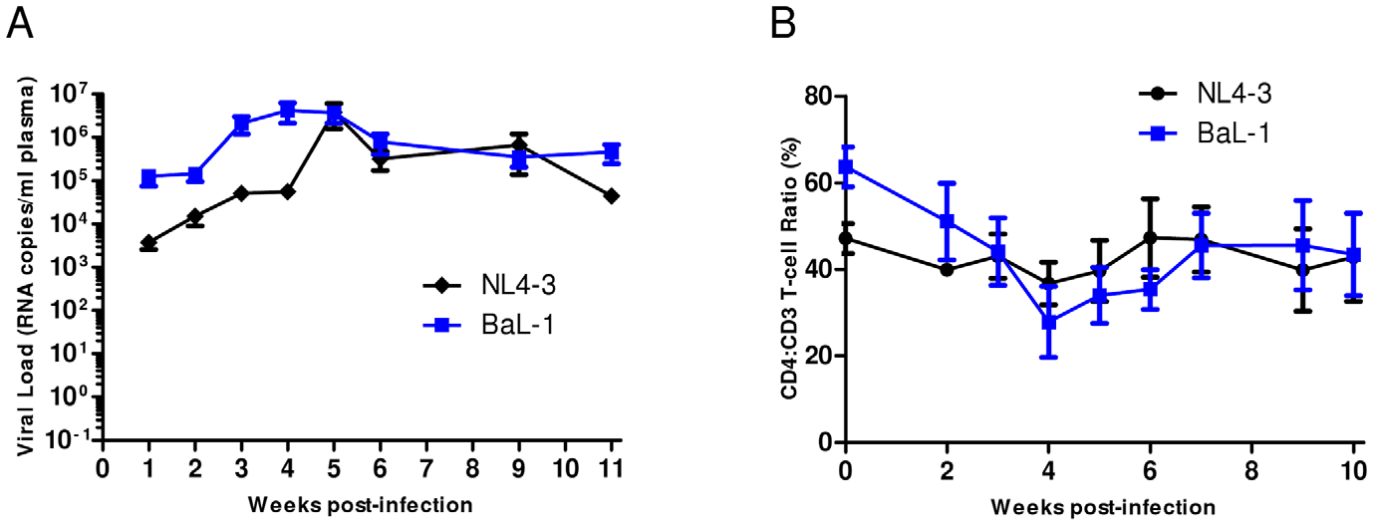
Humanized Rag1^−/−^γc^−/−^ mice are susceptible to HIV-1 infection by i/p route and show CD4 T cell decline. Mice were infected by i/p route with either X4 or R5 HIV-1. Blood was collected weekly and viral RNA extracted from the plasma fraction. Viral RNA loads were determined by Q-RT-PCR as described in methods. Levels of CD4 T cells were monitored on a weekly basis by FACS to determine their decline. Baseline values for each of the mice were established prior to infection as described in Methods. A. RNA viral loads. B. CD4 T cell levels.

We and others have previously shown that humanized Rag2^−/−^γc^−/−^ are susceptible to infection with HIV-1 [19], [20], [26], [35], [36]. Here we evaluated if humanized Rag1^−/−^γc^−/−^ mice also permit infection with both the R5 and X4 tropic HIV-1. Mice were injected with either R5 virus (BaL-1; n = 5) or X4 virus (NL4-3; n = 6) by the i/p route. Infected animals were assayed for viremia by Q-RT-PCR for up to 11 weeks post-infection (Fig. 5A). We found that all virus exposed animals became viremic by the first week post-infection and, as expected, the control uninfected/engrafted animals (n = 3) were negative for any viral RNA. Viremia levels on average peaked at 3.8×10∧6 RNA copies/ml plasma at five weeks post-infection infection and the mice were virus positive throughout the 11 weeks of observation. Since the helper CD4 T cell depletion is a hallmark of HIV infection, their levels in peripheral blood were followed by FACS analysis (Fig. 5B). Our results showed a trend of declining CD4 T cell levels with both of the viruses during the ten week period of evaluation.

**Figure 6:**
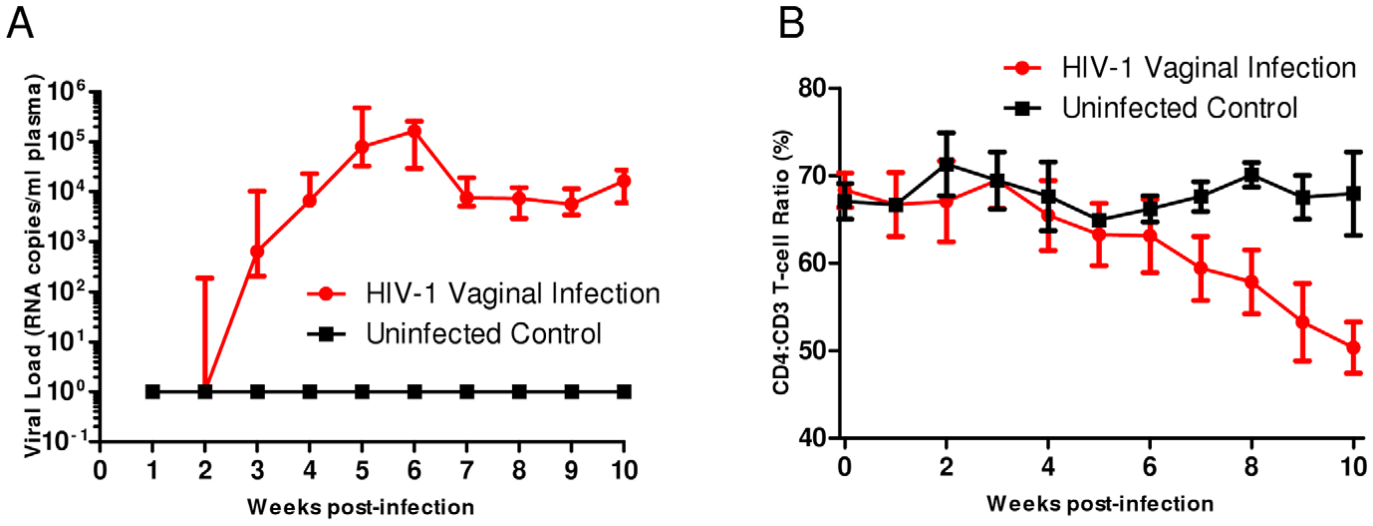
Humanized Rag1^−/−^γc^−/−^ mice are permissive to HIV-1 infection by vaginal route and show CD4 T cell decline. Mice were infected by vaginal route with R5 BaL HIV-1. Blood was collected weekly and viral RNA extracted from the plasma fraction. Viral RNA loads were determined by Q-RT-PCR as described in methods. Levels of CD4 T cells were monitored on a weekly basis by FACS to determine their decline. Baseline values for each of the mice were established prior to infection as described in Methods. A. RNA viral loads. B. CD4 T cell levels.

The above data have shown that humanized Rag1^−/−^γc^−/−^ mice are readily infected by i/p route and have persistent viremia. However, since the most common route of HIV-1 infection is by the heterosexual vaginal route, we asked if these mice are susceptible to infection via this mucosal route as well. Mice were infected vaginally with an R5-tropic HIV-1 which is the predominant strain that is transmitted sexually. Our results showed that all the exposed mice became infected by the second week with the viral loads reaching a peak around 5th week and viremia persisting thereafter (Fig. 6A). Furthermore, the infected mice also exhibited CD4 T cell loss typical to HIV infection (Fig. 6B). Collectively, these data showed that humanized Rag1^−/−^γc^−/−^ mice are permissive for HIV-1 infection by both parenteral and vaginal routes and display the characteristic helper T cell depletion.

## Discussion

Here we have shown that a new strain of immunodeficient mice, Rag1^−/−^γc^−/−^ mice, permits efficient engraftment with human fetal liver derived CD34+ hematopoietic progenitor cells giving rise to multilineage human hematopoiesis. Infection of these mice with HIV-1 leads to chronic viremia and subsequent CD4 helper T cell loss. Furthermore, HIV-1 infection could be established via vaginal route of infection which is the predominant mode of sexual viral transmission. Thus, these studies have further expanded the list of immunodeficient mice that can be used for human cell engraftment and the study of human pathogens infecting the human hematopoietic system.

The multilineage human hematopoiesis in these mice is characterized by the presence of human T cells, B cells, macrophages and dendritic cells which populated different primary and secondary lymphoid organs. We have shown the presence of these cells both by multiparametric FACS analysis and immunohistochemistry. Peripheral blood evaluated at later times showed continued presence of human cells beyond one year (data not shown). Viral challenge data demonstrated that these mice could be readily infected with both R5 and X4 tropic HIV-1 wherein mice showed viremia within a week post infection by i/p route. The viremic mice also showed CD4 T cell loss which is a characteristic feature of HIV-1 infection in the human. Mucosal transmission by the vaginal route constitutes the predominant mode of HIV-1 viral transmission. However, details of viral infection at the portal of entry and systemic spread from there-on are poorly understood. While studies in macaques using SIV have shed some light on these early events [37], a suitable small animal model that employs HIV-1 itself would be more ideal to study these aspects in further detail. In this context, thus far only two humanized mouse models with multilineage hematopoiesis have been shown to be suitable for HIV-1 transmission by the vaginal and/or rectal routes [27], [29], [30], [34]. These consist of humanized BLT mice and Rag2^−/−^γc^−/−^ mice. Here we have shown that humanized Rag1^−/−^γc^−/−^ mice are also permissive for vaginal infection with R5 tropic HIV-1 which is the predominant viral strain that is transmitted sexually thus providing yet another mouse model for viral mucosal transmission. This expands the possibilities for testing novel approaches of anti-HIV microbicides and other pre-exposure prophylactic measures as was done with Rag2^−/−^γc^−/−^ and BLT mice [29], [30], [34]. Furthermore, since long-term engraftment is seen, this permits chronic HIV infection studies such as viral latency and development of drug resistance during prolonged anti-retroviral therapies [38].

A recent report also demonstrated successful engraftment of human hematopoietic cells in Rag1^−/−^γc^−/−^ mice [39]. Cord blood derived CD34 cells were utilized in this study and only T cells and B cells were analyzed in the peripheral blood compartment. In contrast, we used fetal liver derived CD34 cells which are believed to result in improved and longer lasting engraftment due to their more primitive lineage characteristics. Moreover, our present analysis is of a broader scope detecting additional human cell sub-sets such as macrophages and dendritic cells in different organs in addition to demonstrating susceptibility to HIV-1 infection.

Due to their unique nature of providing human hematopoietic cells at various stages of maturation in an in vivo setting, the new humanized mice are likely to play an ever increasing role in biomedical research. In the context of HIV, these mice were effectively used in evaluating novel therapeutic strategies that used peptides, small molecule drugs, vector-expressed antibodies and zinc-finger nucleases, siRNAs, aptamers and aptamer-siRNA chimeric molecules specifically targeted to HIV infected cells in vivo [32], [33], [34], [40], [41], [42], [43], [44], [45]. Intensification and scale-up of such promising studies requires production of these mice in larger numbers for a thorough evaluation. However, production of humanized mice at such a large scale is labor intensive and other important factors are known to come into play. These include efficient breeding among others. In this context, two factors namely, larger litter size and increased survival post-engraftment favorably contributed to the overall higher yield of humanized Rag1^−/−^γc^−/−^ mice as compared to Rag2^−/−^γc^−/−^ mice.

In summary, the new Rag1^−/−^γc^−/−^ mouse strain permits efficient human hematopoietic cell engraftment, is readily susceptible to HIV-1 infection by both i/p and vaginal routes, appears to be generally more robust than Rag2^−/−^γc^−/−^ mice and therefore provides yet another choice to generate humanized mice.
